# *Atlantochodaeus*, A New Genus of Ochodaeidae Streubel, 1846 (Coleoptera, Scarabaeoidea) from Brazilian Atlantic Forest with Four New Species and Morphological Notes to the Family

**DOI:** 10.1007/s13744-025-01288-0

**Published:** 2025-06-17

**Authors:** Vinícius da Costa-Silva, Rafael Sousa, Juares Fuhrmann, Paschoal C. Grossi, Fernando Z. Vaz-de-Mello

**Affiliations:** 1 Invertebrate Biosystematics and Conservation Group, Department of Zoology and Entomology, University of Pretoria, Hatfield, South Africa; 2 Laboratório de Scarabaeoidologia, Instituto de Biociências, Universidade Federal de Mato Grosso (UFMT), Cuiabá, Mato Grosso Brazil; 3 Laboratório de Coleoptera (LAC) do Museu de Zoologia (MZSP), Universidade de São Paulo (USP), São Paulo, São Paulo Brazil; 4 Laboratório de Taxonomia de Insetos, Departamento de Agronomia/Fitossanidade, Universidade Federal Rural de Pernambuco (UFRPE), Recife, Pernambuco Brazil

**Keywords:** Brazil, Morphology, Neotropical, Ochodaeinae, Taxonomy

## Abstract

*Atlantochodaeus*, a new genus of Ochodaeidae from the Brazilian Atlantic Forest is described, discussed, and illustrated. Additionally, four new species belonging to *Atlantochodaeus* are described: *Atlantochodaeus everardoi* **n. sp.** and *Atlantochodaeus paulseni* **n. sp.**, both recorded from Rio de Janeiro State; *Atlantochodaeus hucheti* **n. sp.** from Espírito Santo, Minas Gerais, Rio de Janeiro, and São Paulo States, and *Atlantochodaeus oliviae* **n. sp.** from Minas Gerais State. Detailed descriptions of these species are accompanied by a differential diagnosis and a distribution map. A dichotomous key for all South American genera of Ochodaeidae and the species of *Atlantochodaeus* is provided to aid in identification. A comprehensive comparative morphology study regarding *Parochodaeus pectoralis* (LeConte, 1868) (type species of *Parochodaeus*) and the *Atlantochodaeus* species are discussed and illustrated. A discussion about gland, mycangium and stridulatory apparatus of Ochodaeinae are also provided. These findings emphasize the richness of the Atlantic Forest and underscore the importance of detailed taxonomic studies in revealing its biodiversity.

## Introduction

Ochodaeidae Streubel, 1846 is a widely distributed family of scarab beetles (Coleoptera: Scarabaeoidea) and is easily separate from other scarab by the presence of a crenulation or serration in at least one of both mesotibial spurs (Scholtz and Grebennikov 2005).

The family is divided into two extant subfamilies: Chaetocanthinae Scholtz, 1988 and Ochodaeinae Streubel, 1846. Chaetocanthinae have meso- and metatibial spurs crenulated or pectinate, metatibia somewhat flat, and abdomen without stridulatory apparatus; otherwise, Ochodaeinae have mesotibial spur crenulated or pectinate, metatibial spurs not crenulated or pectinated, metatibia not flat, and abdomen usually with stridulatory apparatus. The last subfamily is divided in three tribes (Bouchard et al. 2024): Endognathini Scholtz, 1988 (with *Endognathus* Benderitter, 1920 and *Odontochodaeus* Paulian, 1976 from Madagascar), Nothochodaeini Nikolajev 2015 (*Nothochodaeus* Nikolajev, 2005, *Mimochodaeus* Nikolajev 2009 and *Ceratochodaeus* Huchet, 2017 from Asia), and Ochodaeini. Endognathini is separate from other tribes by its large head, mandibles and pedicels, and the thin and long scutellum with posterior angle acuminated. Nothochodaeini is separated mainly by the mesocoxae widely separated from each other, elytral interlocking apparatus of propygidium with trapezoid carina, and female genitalia without styli. Otherwise, Ochodaeini has head, mandibles and pedicel relatively small (as Nothochodaeini), mesocoxae usually subcontiguous (as Endognathini), elytral interlock apparatus of propygidium variable (absent, with two posterior small spines, with two longitudinal parallel or posteriorly convergent carina, or with posterior margin upturned), and female with styli (as Endognathini) (Scholtz et al. 1988; Huchet 2017, 2019, 2021; Nikolajev 2005, 2015).

Including the results presented here, Ochodaeini Streubel, 1846 now includes eight extant genera: *Afrochodaeus* Huchet 2020 [Afrotropical region]; *Atlantocodaeus* Costa-Silva, Sousa, Fuhrmann, Grossi and Vaz-de-Mello, **new genus** [Neotropical]; *Codocera* Eschscholtz, 1821 [Nearctic]; *Cucochodaeus* Paulsen 2007 [Nearctic]; *Neochodaeus* Nikolajev 1995 [Nearctic, Neotropical]; *Ochodaeus* Dejean, 1821 [Afrotropical, Palaearctic, Neotropical, Oriental, and Madagascan]; *Parochodaeus* Nikolajev 1995 [Afrotropical, Nearctic, Neotropical, and Palearctic]; and *Xenochodaeus* Paulsen 2007 [Nearctic] (Nikolajev 2009; Paulsen 2012; Huchet 2016; Schoolmeesters 2024). These beetles are primarily active during the night and are often collected using light traps, including those emitting white light or UV, as well as flight interception traps (Scholtz et al. 1988; Ratcliffe and Paulsen 2008; Paulsen and Ocampo 2012).

Among the aforementioned genera, the genus *Parochodaeus* comprises approximately 27 species that range from the USA to Argentina (Arrow 1912; Paulsen 2011; Paulsen and Ocampo 2012; Schoolmeesters 2024), with one additional species occurring from Europe [*Parochodaeus pocadioides* (Motschulsky, 1859) in Portugal and Spain, see Huchet 2016] and other from Afrotropical/Palearctic [*Parochodaeus carinatus* (Benderitter, 1913) in Somalia, Ethiopia, Kenya, Djibouti, Yemen and Oman; see Huchet 2020]. Prior to present study, this genus was the only representative of Ochodaeinae in South America and includes 13 species reported to the continent (Paulsen and Ocampo 2012; Paulsen 2012). The most conspicuous morphological characteristic of *Parochodaeus* is the presence of an elytral interlocking apparatus of pygidium formed by two tubercles on the posterior margin that interlock with the sharp apices of the elytra (for additional characteristics, see Paulsen 2011 and Paulsen and Ocampo 2012). The species of genus *Parochodaeus* are typically found in arid and sandy habitats, although there are records of species occurring in forested areas as well (Paulsen and Ocampo 2012).

The sole research conducted on Ochodaeidae fauna in South America was carried out a decade ago by Paulsen and Ocampo (2012), with a specific focus on the Argentine fauna. The study described the genus *Gauchodaeus* (the unique Chaetocanthinae from South America) and seven species of *Parochodaeus*, most of them exclusively documented for Argentina, and reported the occurrence of *Parochodaeus campsognathus* (Arrow 1904) and *P*. *cornutus* (Ohaus 1910) to Brazil.

Until now, three species of Ochodaeidae have been reported in Brazil (Vaz-de-Mello and Costa-Silva 2024): *Parochodaeus jatahyensis* (Benderitter 1912), known solely from its type locality in Jataí, Goiás State (GO); *P*. *campsognathus*, recorded in Mato Grosso State (MT) and Rio Grande do Sul State (RS); and *P*. *cornutus*, documented exclusively in Pelotas, RS (Paulsen and Ocampo 2012; Vaz-de-Mello and Costa-Silva 2024). Apart from the southern (RS) and Mid-west region (GO and MT), no other records of *Parochodaeus* or other genera within Ochodaeidae have been reported in Brazil, underscoring the lack of information and limited recognition of the group’s biodiversity on a national scale (Vaz-de-Mello and Costa-Silva 2024).

The goal of the present paper is to describe four new species of Ochodaeini that are, so far, only known to the Brazilian Atlantic Forest, and a new genus *Atlantochodaeus* to accommodate these species. *Parochodaeus* resembles the new genus, and a comparative diagnosis is added. Differential diagnosis for the new species, as well as a map illustrating their geographical distribution are added, and discussion of some morphological characteristics to Ochodaeinae are also presented.

## Material and Methods

A total of 88 specimens belonging to Ochodaeini were studied. The collections/depositories of each specimen examined is listed below. Curators are given in brackets. **AMBC**Ayr Moura Bello private collection, Rio de Janeiro, Brazil (Ayr M. Bello); **CEIOC** Coleção Entomológica do Instituto Oswaldo Cruz, Rio de Janeiro, Rio de Janeiro, Brazil (Márcio Felix); **CEMT**Coleção Entomológica de Mato Grosso Eurides Furtado, Coleção Zoológica da Universidade Federal de Mato Grosso), Cuiabá, Mato Grosso, Brazil (Fernando Z. Vaz-de-Mello); **CERPE**Coleção Entomológica da Universidade Federal Rural de Pernambuco, Recife, Pernambuco, Brazil (Paschoal C. Grossi); **MZSP**Museu de Zoologia, Universidade de São Paulo, São Paulo, Brazil (Sônia A. Casari).

Photographs were captured using a Canon EOS 80D DLSR camera using a Canon MP-E 65 mm f/2.8 macro-lens. Line drawings were produced using camera lucida attached to a Carl Zeiss Stemi SV6 stereomicroscope (magnification: 10–50x) and a Carl Zeiss Axioskop microscope (magnification: 100–400x). Maps were developed with the assistance of QGis for Desktop version 3.16.9 software. All plates were edited using Adobe Photoshop CC 2019 software.

Label data for the type specimens are transcribed verbatim within quotation marks “…”, and handwritten information is underlined. A slash bar “/” is used to indicate a new line of text on the same label. Explanatory information on the labels is indicated with square brackets “[]”. All examined specimens were cleaned following the protocols outlined by Costa-Silva and Diéguez (2020). Detached parts were card mounted with acid-free, water-soluble glue.

The terminology used follows Scholtz and Grebennikov (2005) as summarized in Costa-Silva et al. (2024) and Harris (1979) for the surface sculpturing.

## Results

### Taxonomy

**Family** Ochodaeidae Streubel, 1846

**Subfamily** Ochodaeinae Streubel, 1846

**Tribe** Ochodaeini Streubel, 1846

**Genus*** Atlantochodaeus* **n. gen.**

*Atlantochodaeus* Costa-Silva, Sousa, Fuhrmann, Grossi and Vaz-de-Mello, **n. gen.**

(Figs. 1, 2a–g, 3e–l, 4, 5, 6, 7, 8, 9, 10, 11, 12, 13).

**Fig. 1:**
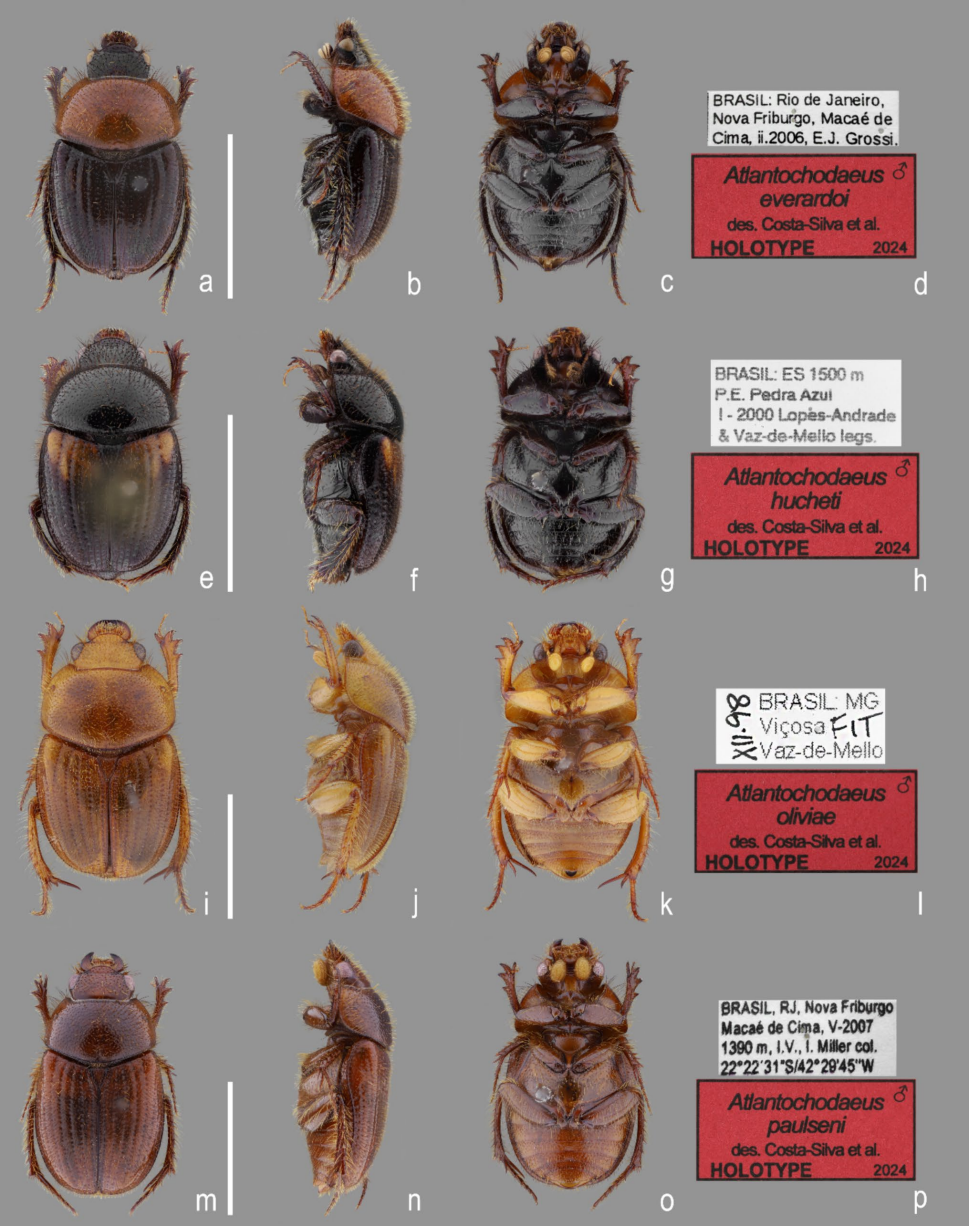
*Atlantochodaeus* **new genus**, holotypes (dorsal, lateral, ventral, labels). **a**–**d**, *A*. *everardoi* **new species**. **e**–**h**, *A*. *hucheti* **new species**. **i**–**l**, *A*. *oliviae* **new species**. **m**–**p**, *A*. *paulseni* **new species**. Scale = 5 mmss

**Fig. 2:**
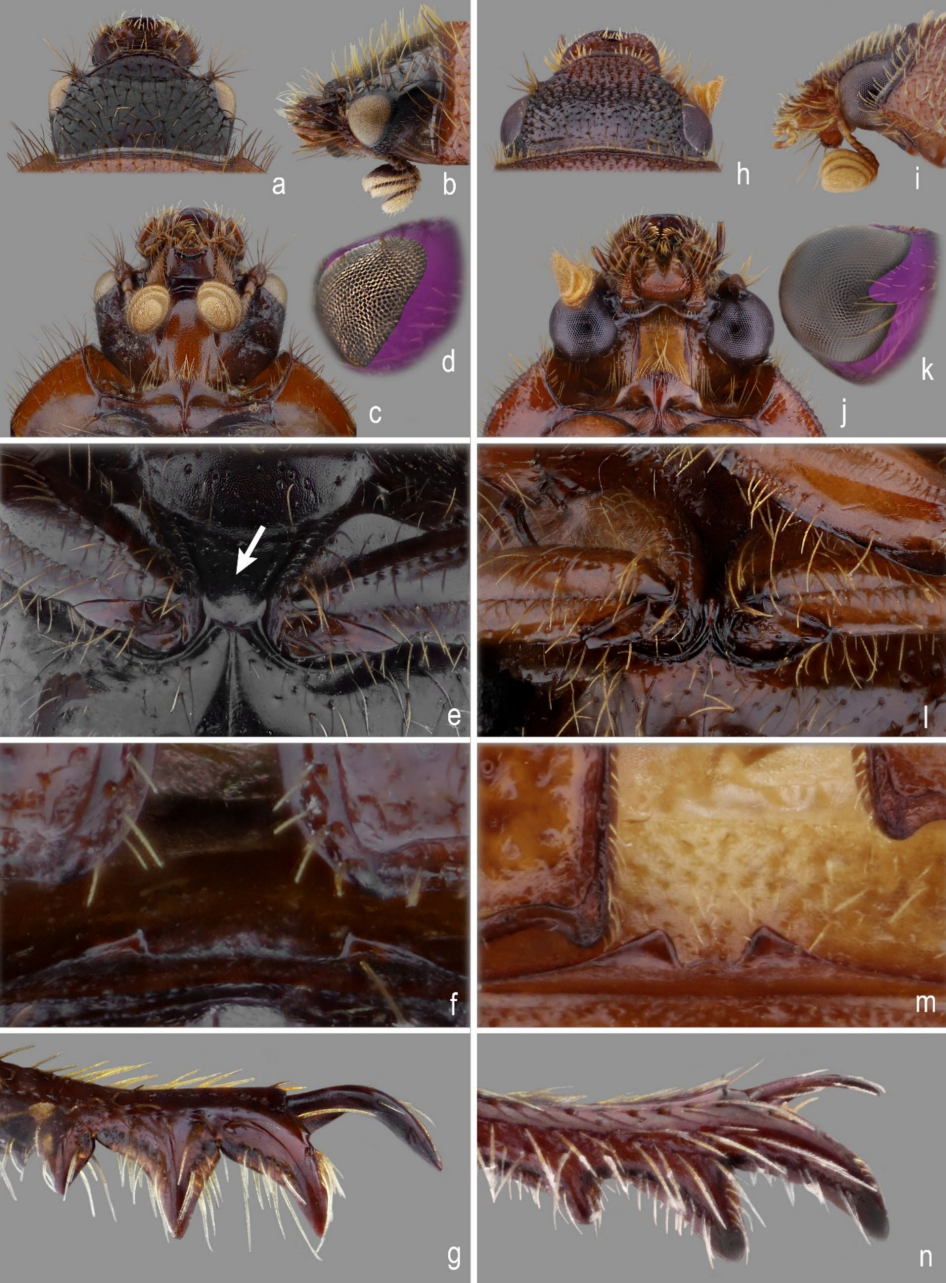
Ochodaeidae morphology. **a**–**g**, *Atlantochodaeus everardoi* **new genus and species**, male. **h**–**n**, *Parochodaeus pectoralis* (LeConte, 1868), male. **a**, **h**, head, dorsal view. **b**, **i**, head, lateral view. **c**, **j**, head, ventral view. **d**, **k**, eye posterolateral margin. **e**, **l**, mesocoxae (arrow pointing coxae separation). **f**, **m**, detail of propygidium-elytra interlocking apparatus. **g**, **n**, protibia

**Fig. 3:**
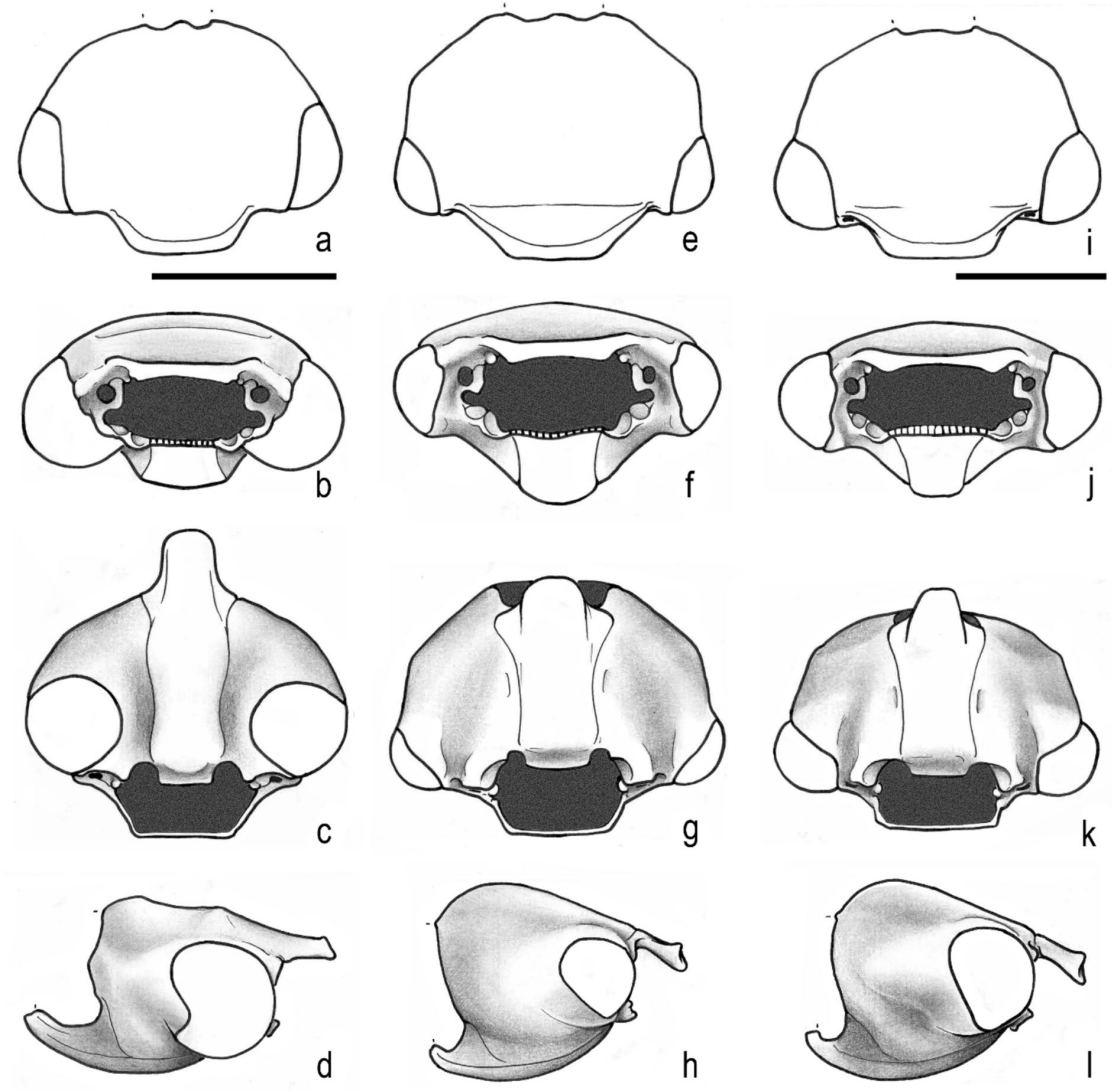
Head (dorsal, frontal, ventral, lateral). **a**–**d**, *Parochodaeus pectoralis* (LeConte, 1868). **e**–**h**, *Atlantochodaeus everardoi* **new species**. **i**–**l**, *A*. *oliviae* **new species**. Scale = 1 mm

**Fig. 4:**
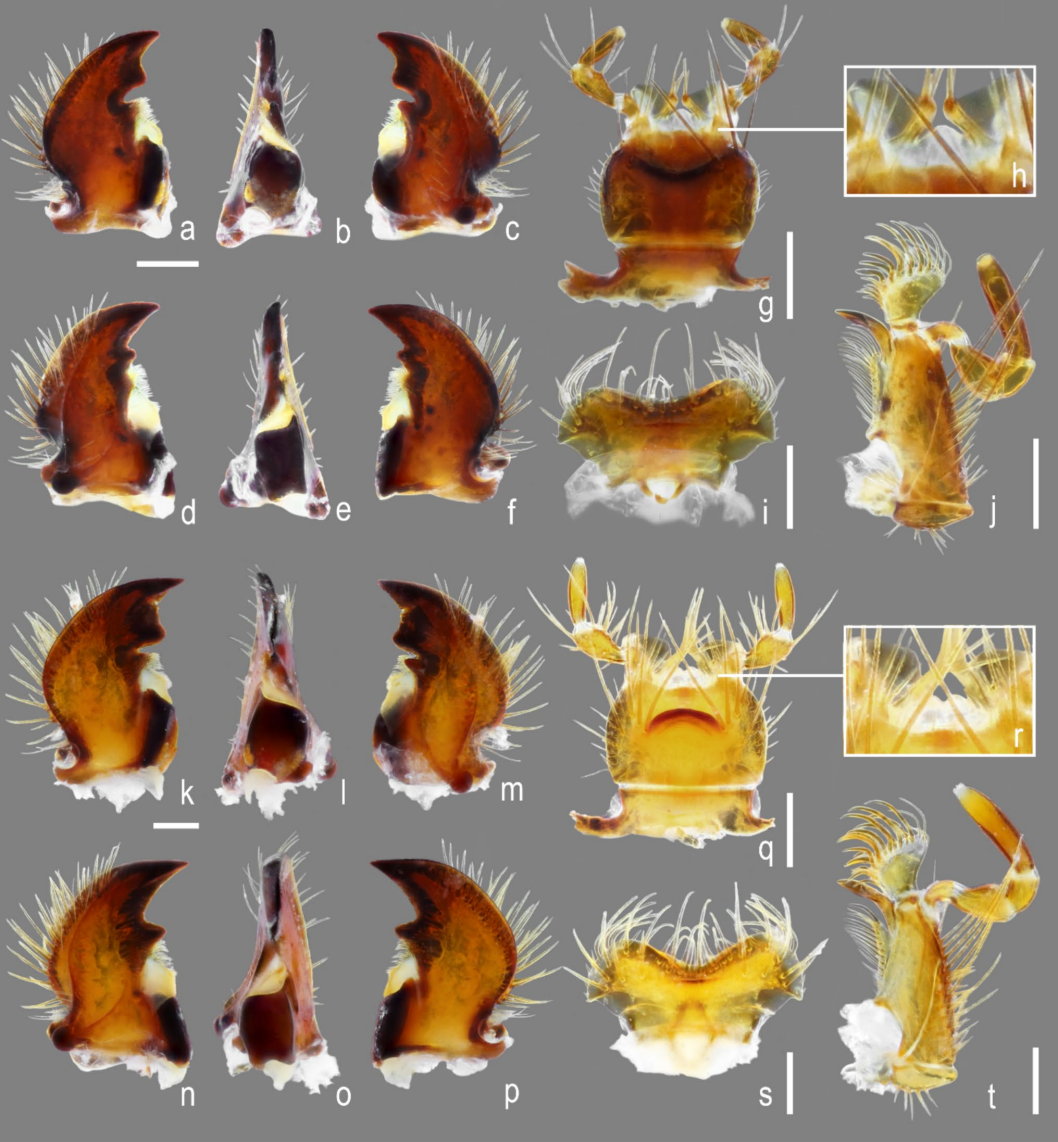
*Atlantochodaeus* **new genus**, mouthparts. **a**–**j**, *A*. *everardoi* **new species**. **k**–**t**, *A*. *oliviae* **new species**. **a**–**c**, **k**–**m**, left mandible (dorsal, inner, ventral). **d**–**f**, **n**–**p**, right mandible (ventral, inner, dorsal). **g**, **q**, labium (ventral). **h**, **r**, Detail of anterior area of prementum. **i**, **s**, labrum (dorsal). **j**, **t**, maxilla (ventral). Scale = 0.5 mm; details without scale

**Fig. 5:**
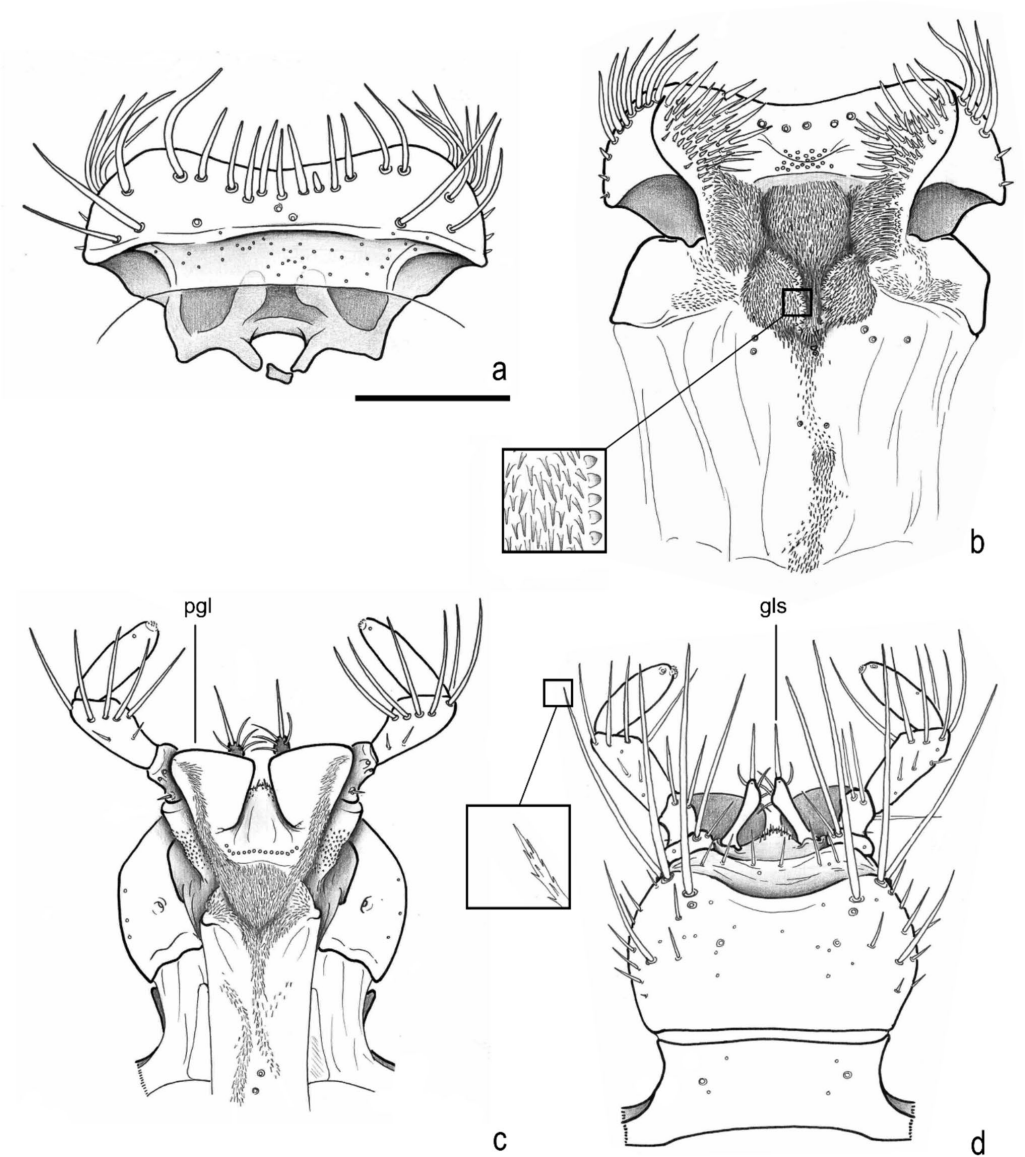
*Atlantochodaeus everardoi* **new genus and species**, mouthparts. **a**, labrum (dorsal). **b**, epipharynx (ventral, with detail of asperites). **c**, labium and hypopharynx (dorsal). **d**, labium (ventral, with detail of setae). **pgl**, paraglossa; **gls**, glossa. Scale = 0.5 mm

**Fig. 6:**
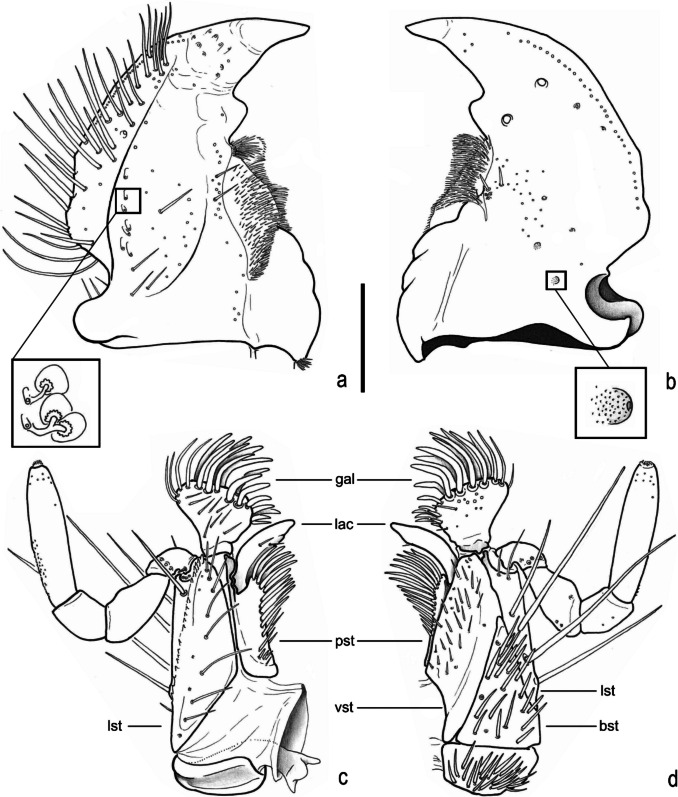
*Atlantochodaeus everardoi* **new genus and species**, mouthparts. **a**, **b**, right mandible (ventral with details of internal morphology, dorsal with detail of small fovea). **c**, **d**, maxilla (dorsal, ventral, respectively). **bst**, basistipe; **gal**, galea; **lac**, lacinia; **lst**, laterostipe; **pst**, parastipe; **vst**, ventrostipe. Scale = 0.5 mm

**Fig. 7:**
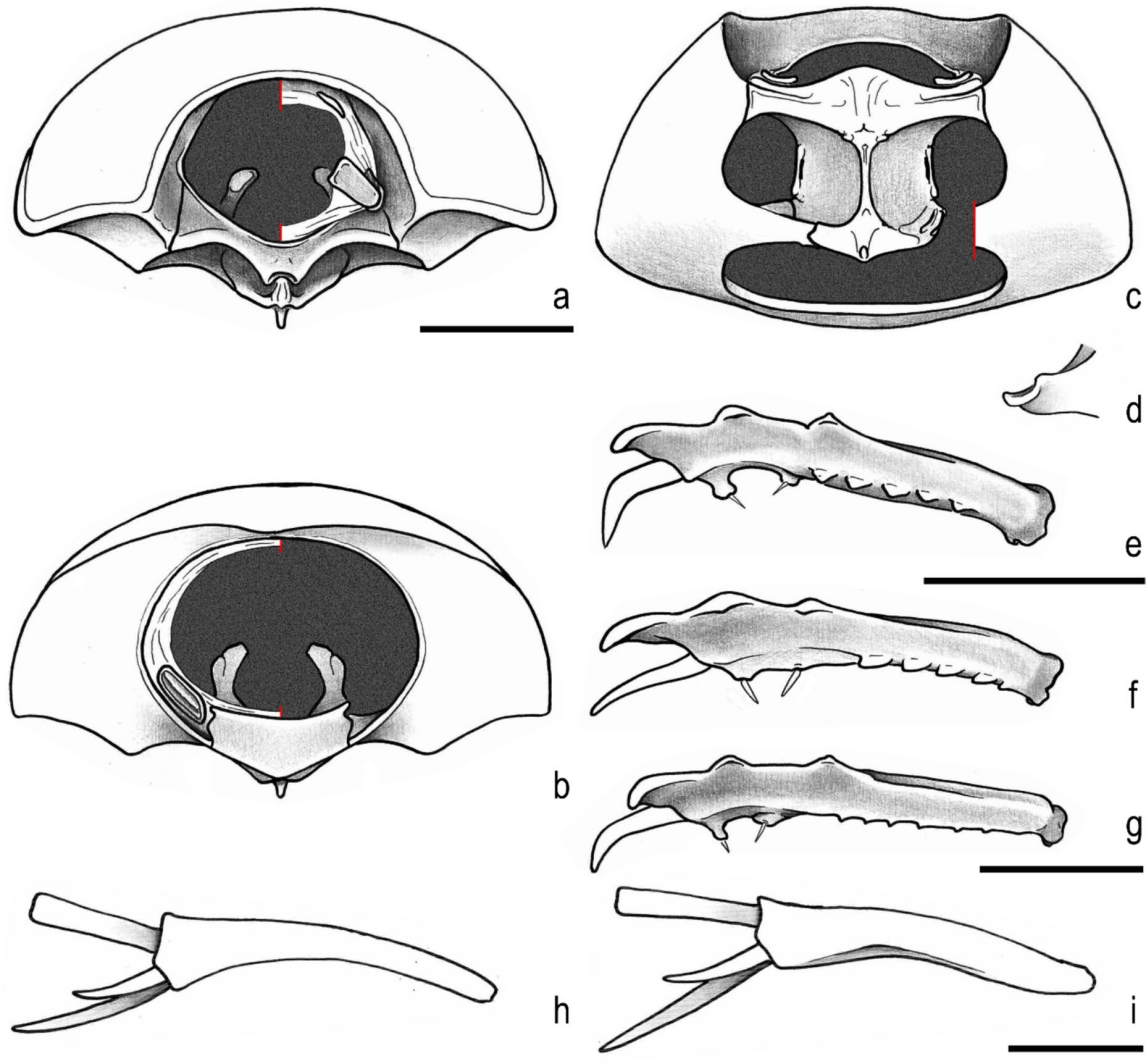
*Atlantochodaeus* **new genus**, prothorax and legs. **a–f**, **h**, *A*. *everardoi* **new species**. **g**,** i**, *A*. *oliviae* **new species**. **a**, prothorax, frontal (left side with cervix); **b**, prothorax, posterior (left side with intersegmentar membrane); **c**, prothorax, ventral (left side with arm of hypomeron dissected); **d**, detail of ornaments of posterior arm of hypomeron; **e–g**, protibia, outer view (male, female, male); **h**, **i**, metatibia posterior view. Scale = 1 mm

**Fig. 8:**
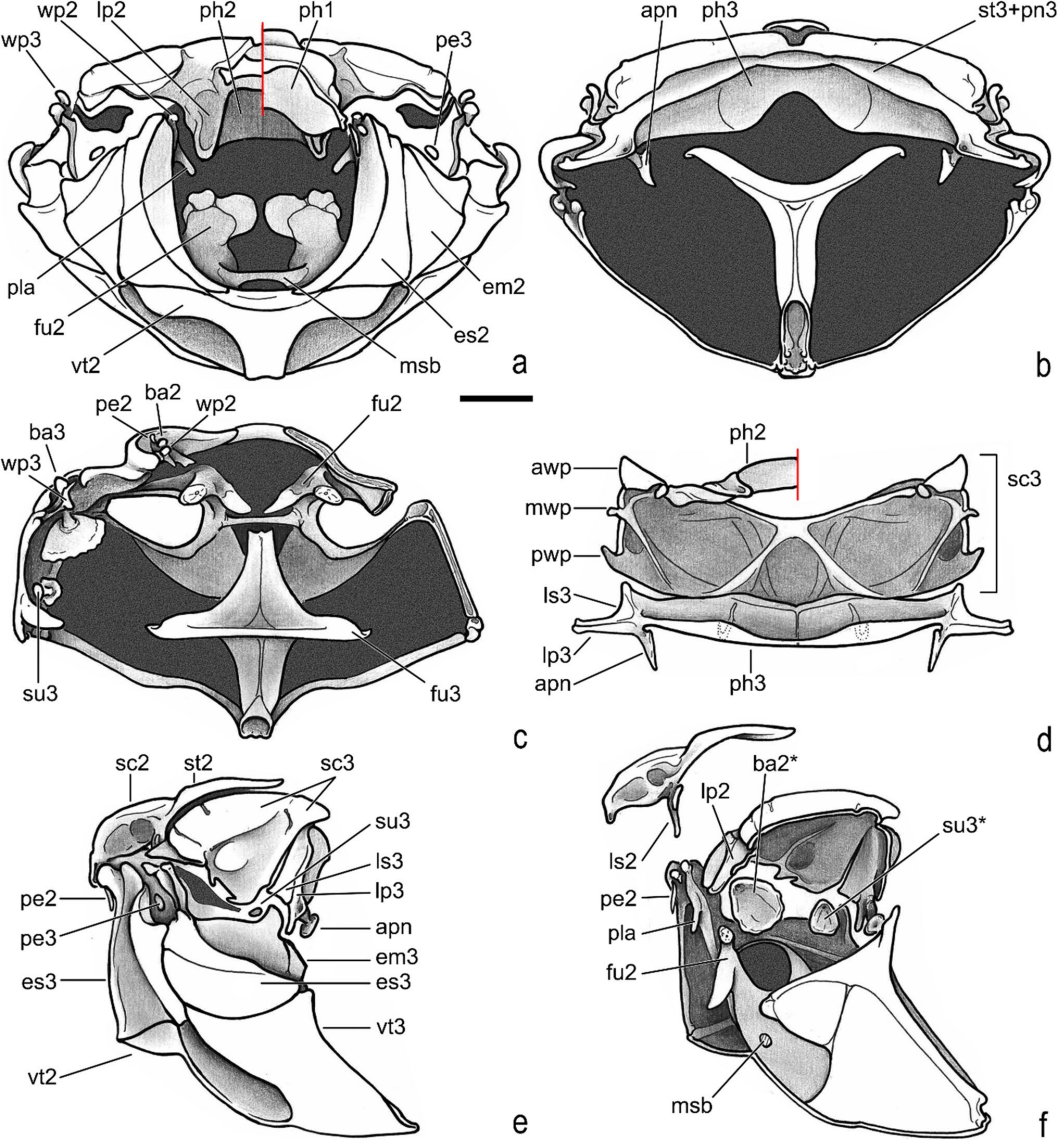
*Atlantochodaeus everardoi* **new genus and species**, pterothorax. **a**, anterior (right side with mesoalinotum omitted). **b**, posterior. **c**, dorsal, internal (right side with epimesterna and epimera ommited). **d**, ventral, internal (left side with mesopostnoum elements omitted). **e**, **f**, lateral (external, internal with mesoalinotum setarate). **Acronyms suffix 2 and 3** indicating mesothorax and metathorax elements, respectively (except to fragma: **ph1–3**); **apn**, apodeme of postnotum; **awp**, anterior notal wing process; **ba2–3**, basalare (* apodeme); **em2–3**, epimeron; **es2–3**, episternum; **fu2–3**, furca; **lp2–3**, lateral arm of postnotum; **ls2–3**, lateral arm of scutellum; **msb**, mesendosternite bridge; **mwp**, medial notal wing process; **pe2–3**, prealare; **ph1–3**, first, second and third phragma; **pla**, pleural arm; **pwp**, posterior notal wing process; **sc2–3**, scutum; **su3**, subalare (* apodeme); **st2–3**, scutellum; **vt2–3**, meso- and metaventrite; **wp2–3**, pleural wing process. Scale = 1 mm

**Fig. 9:**
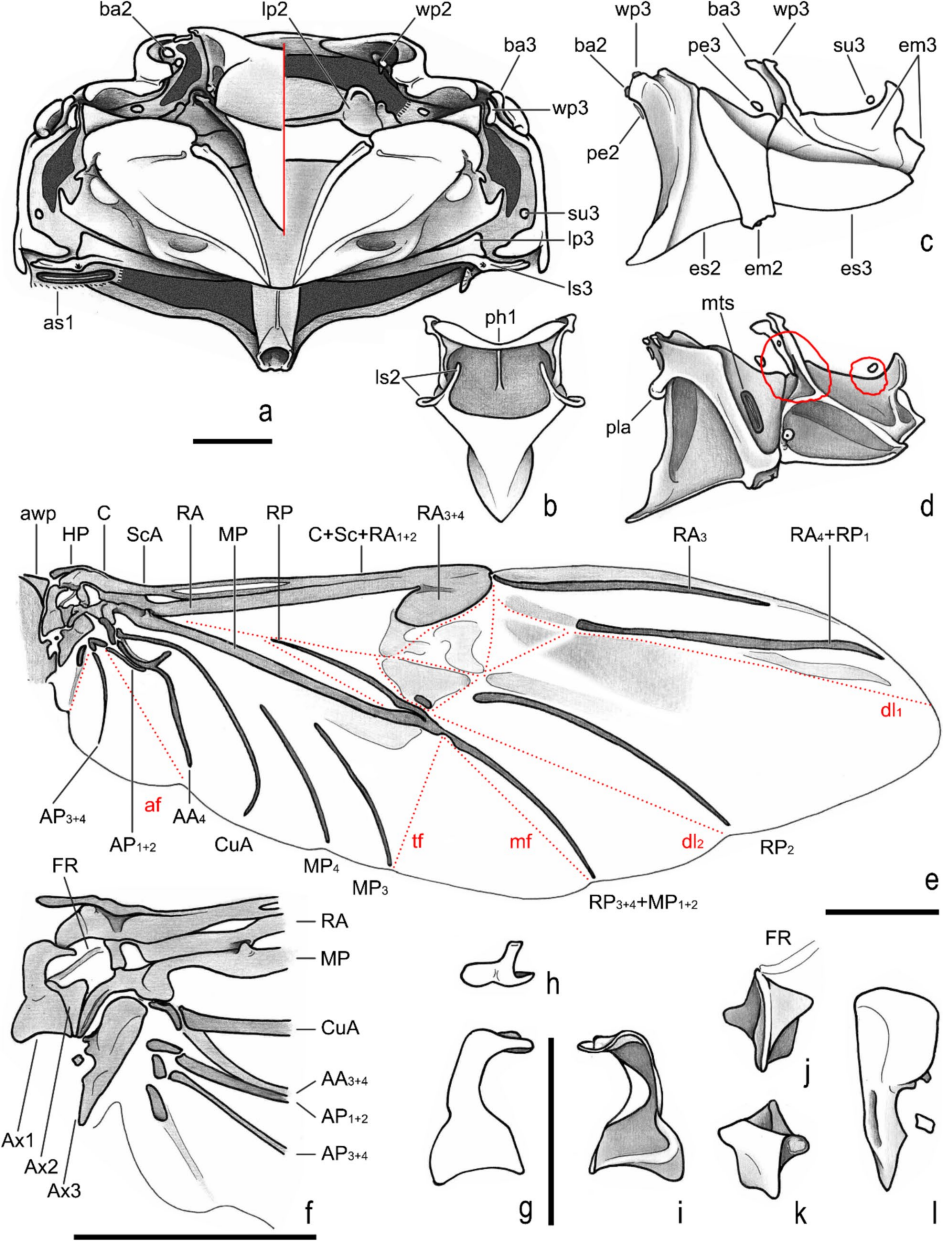
*Atlantochodaeus everardoi* **new genus and species**, pterothorax. **a**, dorsal (right side with mesoalinotum omitted). **b**, mesoalinotum, internal, ventral. **c**, **d**, pleurites (external, internal with margins of apodemes of basalare and suralare in red). **e**, right posterior wing. **f**, detail of posterior wing basis. **g–i**, first axillary sclerite (head, dorsal, ventral, respectively). **j**, **k**, second axillary sclerite (dorsal with radial fulcalare associated, ventral). **l**, third axillary sclerite, dorsal. **Acronyms suffix 2 and 3** to thorax indicating mesothorax and metathorax elements, respectively (except to axillary sclerites and phragma, Ax1–3, **ph1**). **awp**, anterior notal wing process; **ba2–3**, basalare; **em2–3**, epimeron; **es2–3**, episternum; **lp2–3**, lateral arm of postnotum; **ls2–3**, lateral arm of scutellum; **mts**, metathoracic spiracle; **pe2–3**, prealare; **ph1**, first phragma; **pla**, pleural arm; **su3**, subalare; **wp2–3**, pleural wing process. **Veins** (anterior to posterior): **C**, costa; **Sc**, subcosta; **RA**, radius anterior; **RP**, radius posterior; **MP**, medial posterior; **CuA**, cubital anterior; **AA**, anal anterior; **AP**, anal posterior. **Folds** (dotted lines; anterior to posterior): **dl1**, **dl2**, longitudinal fold 1 and 2; **mf**, medial fold; **tf**, transversal fold; **af**, anal fold; **jf**, jugal fold. Scale: **A–F** = 1 mm; **G–L** = 0.5 mm

**Fig. 10:**
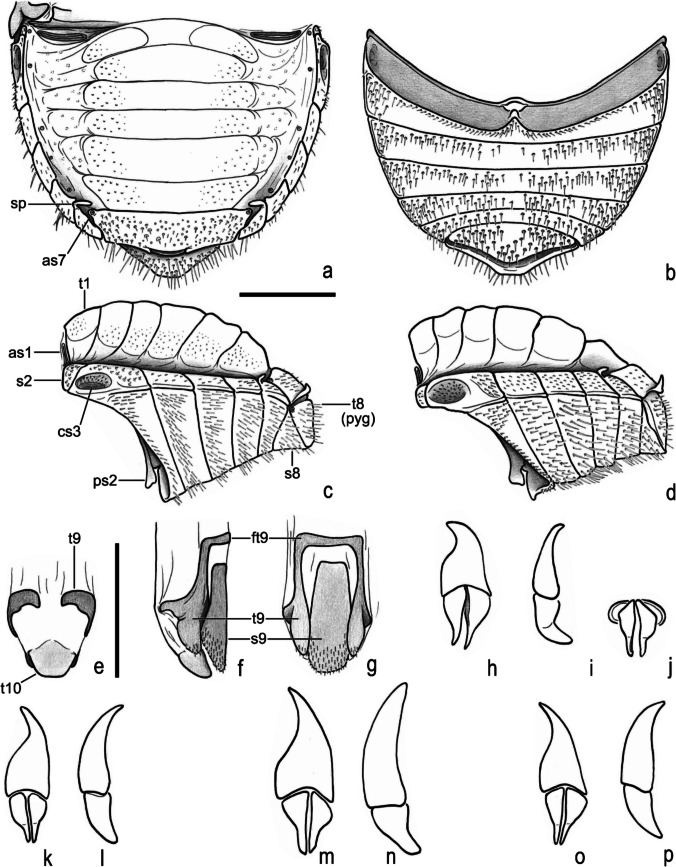
Ochodaeidae, abdomen. **a–c**, **e–j**, *Atlantochodaeus everardoi* **new species**. **d**, *Parochodaeus pectoralis* (LeConte, 1868). **k**, **l**, *Atlantochodaeus hucheti* **new species**. **m**, **n**, *A*. *oliviae* **new species**. **o**, **p**, *A*. *paulseni* **new species**. **a–d**, abdomen (dorsal, ventral, lateral, and lateral view, respectively). **e–g**, genital ring. **h**, **k**, **m**, **o**, aedeagus, dorsal. **i**, **l**, **n**, **p**, aedeagus, lateral. **j**, aedeagus, posterior. **as1–7**, abdominal spiracle I**–**VII; **cs3**, lateral concavity of sternite III; **ft9**, ventral fold of tergite IX; **ps2**, medial process of sternite II; **pyg**, pygidium; **s2–9**, sternite II–IX; **sp**, sternite stridulatory peg; **t1–10**, tergite I–X. Scale = 1 mm

**Fig. 11:**
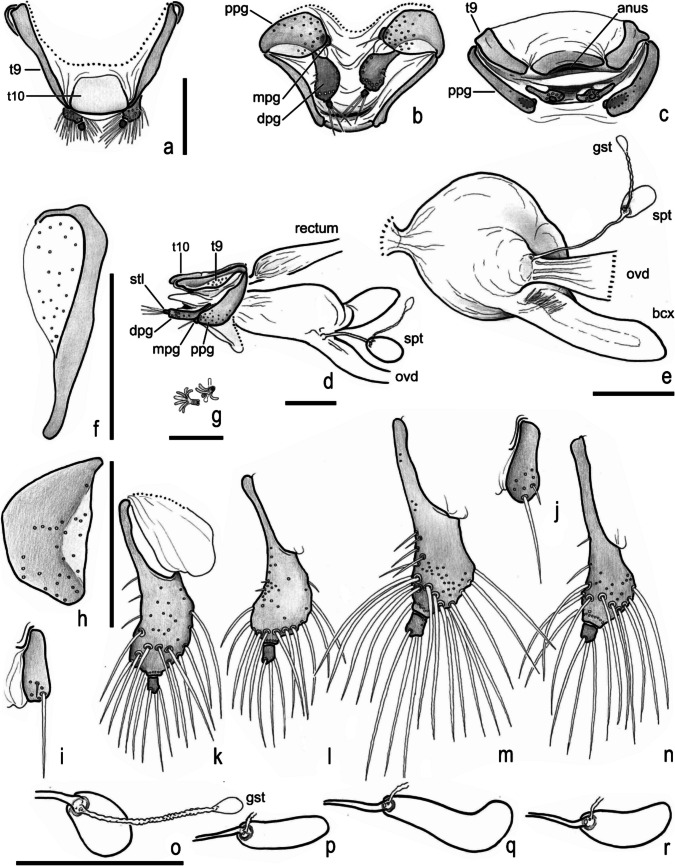
*Atlantochodaeus* **new genus**, female terminalia. **a**–**i**, **k**, **o**, *A*. *everardoi* **new species**. **l**, **p**, *A*. *hucheti* **new species**. **j**, **m**, **q**, *A*. *oliviae* **new species**. **n**, **r**, *A*. *paulseni* **new species**. **a–d**, terminalia (dorsal, ventral, posterior, lateral). **e**, internal genitalia, ventral. **f**, left paraprocts (tergite 9). **g**, internal structure of puncture of paraprocts and proximal gonocoxite. **h**, left proximal gonocoxite. **i**, **j**, left medial gonocoxite. **k–n**, left distal gonocoxite and gonostyle. **o–r**, spermatheca. **bcx**, bursa copulatrix; **dpg**, distal gonocoxite; **gst**, gland of spermatheca; **ovd**, oviduct; **mpg**, medial gonocoxite; **ppg**, proximal gonocoxite; **spt**, spermatheca; **stl**, gonostyle; **t9**, paraproct or abdominal tergite IX; **t10**, proctiger or abdominal tergite 10. Scale: **a–f**, **h–r** = 0.5 mm; **g** = 0.1 mm

**Fig. 12:**
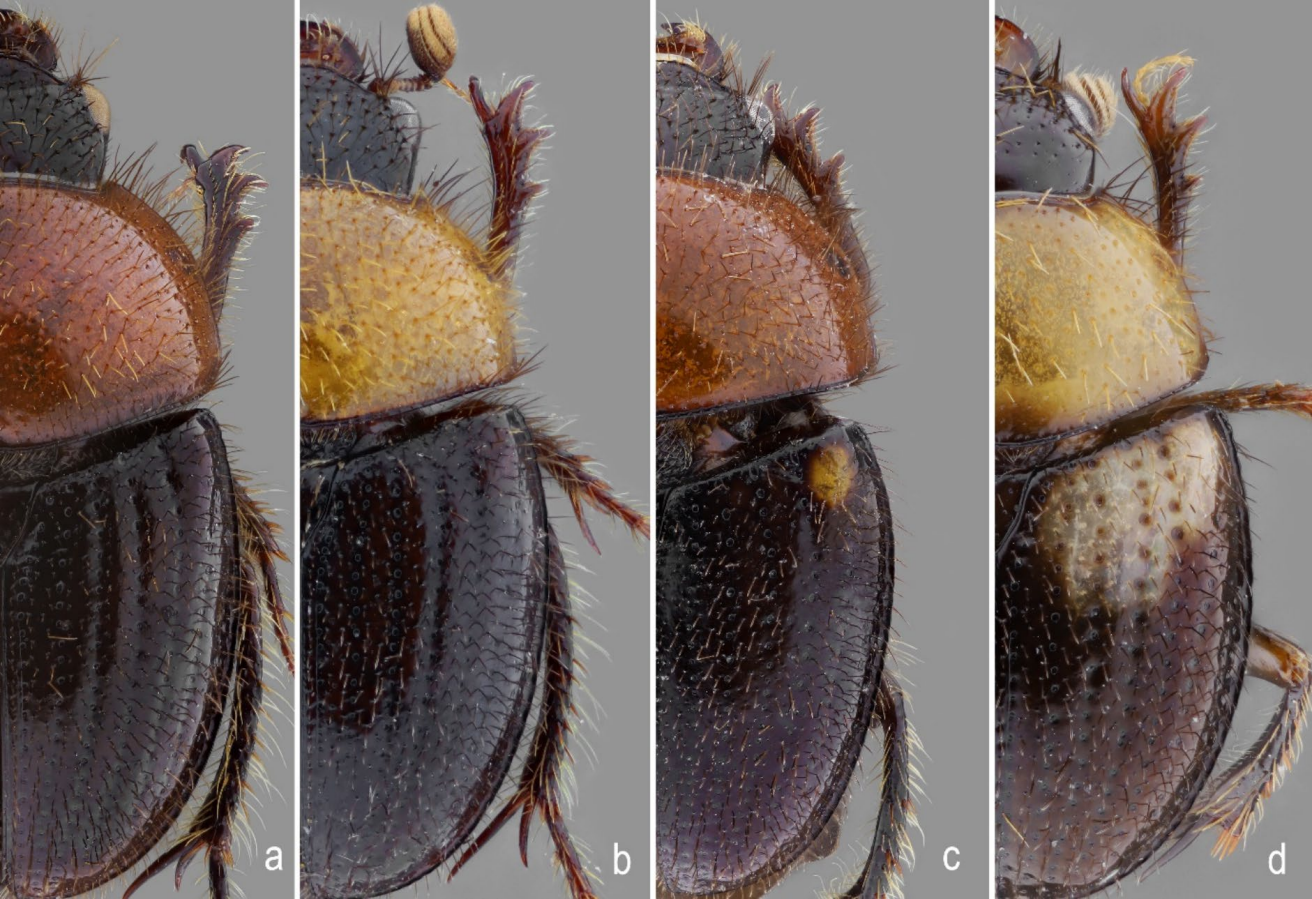
*Atlantochodaeus everardoi* **new genus and species**, colour variation: (**a**) orange pronotum and black elytra; (**b**) yellow pronotum and black elytra; (**c**) orange pronotum and black elytra with a yellow spot in the humeral region; and (**d**) yellow pronotum and black elytra with yellow-milk colour band in the humeral region. Colour variation non-sex related

**Fig. 13:**
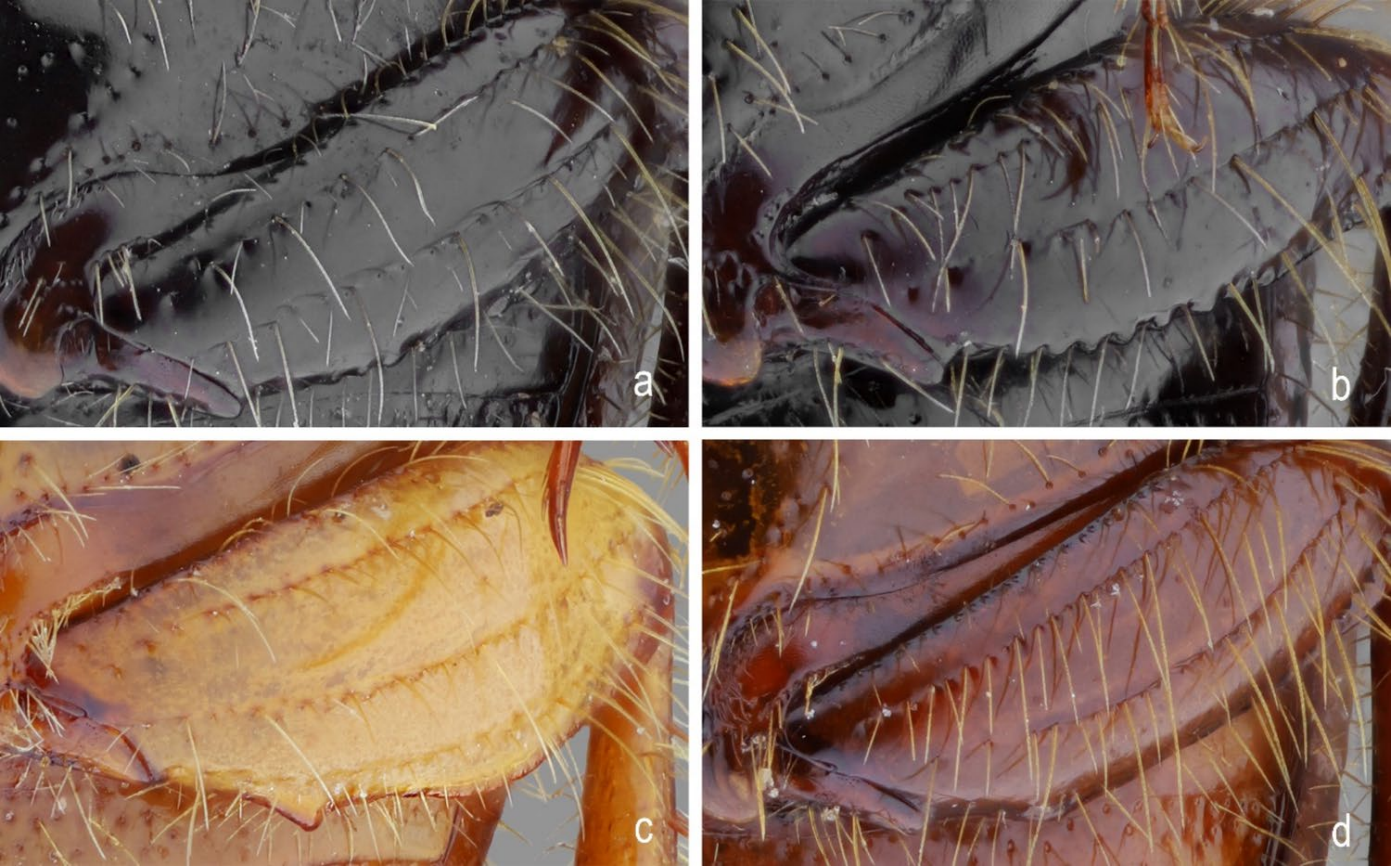
*Atlantochodaeus* **new genus**, metafemur. **a**, *A*. *everardoi* **new species**. **b**, *A*. *hucheti* **new species**. **c**, *A*. *oliviae* **new species**. **d**, *A*. *paulseni*** new species**

(urn:lsid:zoobank.org:act:F551 FDA9-1B76-433 C-A3 AF-C4BCC1E19D3B).

#### **Generic differential diagnosis**

The *Atlantochodaeus* can be separate from the genus *Parochodaeus* by the following characters (*Parochodaeus pectoralis* morphology between square brackets): latero-posterior border of eyes round (Fig. 2d) [emarginated, Fig. 2k]; ventral surface of eye small (Figs. 2c, 3f–h, j–l) [large, Figs. 3b–d]; gula not or slightly prominent posteriorly (Fig. 3h, l) [greatly prominent posteriorly, Fig. 3d]; mesocoxae widely separate (Fig. 2e) [subcontiguous, Fig. 2l]; apex of elytra slightly prominent (Fig. 2f) [strongly prominent, Fig. 2m]; longest metatibial spur as long as or longer than metatarsomere I [shorter]; metatarsomere I straight [usually sinuous] (see Table 1).

**Table 1:** Comparative table among *Parochodaeus pectoralis* and the species of the *Atlantochodaeus* **n. gen**

	*P. pectoralis*	*A. everardoi* **sp. n**	*A. hucheti* **sp. n**	*A. oliviae* **sp. n**	*A. paulseni* **sp. n**
Ventral size of eyes	large	very small	very small	small	small
Posterolateral eye margin	emarginate	rounded	rounded	rounded	rounded
Mentum ventral keel: apex in ventral view	straight	U-shaped	U-shaped	Inverted U-shaped	U-shaped
Prementum, anterior area	lobed	lobed	lobed	emarginate	lobed
Distal angle of elytra	Strongly prominent	weakly prominent	weakly prominent	weakly prominent	weakly prominent
Male protibia, posterior side, inner distal teeth	indistinct	evident	evident	evident	evident
Male protibia, spur	slightly and gradually bent	strongly and abruptly bent	strongly and abruptly bent	strongly and gradually bent	strongly and gradually bent
Mesocoxae	subcontiguous	widely separate	widely separate	widely separate	widely separate
Metafemora	very narrow	narrow	narrow	wide	narrow
Metafemora, inner margin	smooth	weakly crenulate	strongly crenulate	toothed	with small lobe
Metatibia, biggest spur	shorter than tarsomere I	longer than tarsomere I	longer than tarsomere I	longer than tarsomere I	longer than tarsomere I
Metatarsomere I	sinuous	straight	straight	straight	straight
Pygidium, anterior sulcus	absent	present	present	present	present

#### **Description**

Body light or dark yellowish brown (*A*. *olivae* and *A*. *paulseni*), or black with pronotum black (*A*. *hucheti*), orange or yellow (*A*. *everardoi*), humerus with or without light macula. *Head* (Figs. 2a–c, 3e–l): Surface flat, without tubercles or carina, densely punctate and setose, fronto-clypeal suture distinct, complete or indistinct medially, slightly curved (Fig. 3a, i). Gula slightly prominent posteriorly (Fig. 3h, l). Eyes (Fig. 3e–l) prominent, small in dorsal view (head 1.4–1.6 wider than minor dorsal interocular distance; Figs. 2a, 3e, i), small in ventral view (head 1.4–1.6 wider than minor ventral interocular distance; Figs. 2c, 3g, k); posterior margin round (Figs. 2d, 3h, l). Clypeus wider than long, flat; anterior margin rounded, thickened with row of long yellow setae. Labrum (Figs. 4i, s, 5a) slightly sinuous, with strong transversal carina delimiting the anterior and posterior area. Epipharynx (Fig. 5b) with distal medial area with row of about five large sensilla, medial area with about 20 small sensilla, posterolateral areas and posteromedial area with dense comb of cuticular setae-like projections and some asperites. Mandibles (Figs. 4a–f, k–p, 6a–b) slightly asymmetric; outer margin continuously rounded; two large incisor teeth present; mola almost flat and smooth; prostheca densely setose; dorsal proximal area of incisivus with two large punctures. Maxillae (Figs. 4j, t, 6c, d) with stipe divided in four free plates: basistipes (bst), ventrostipes (vst), laterostipe (lst) and parastipe (pst); laterostipes with longitudinal row of minute spines and some long setae; lacinia (lac) falciform and articulated with parastipes; galea (gal) somewhat trapezoid with some thick bent setae; palpus with four palpomeres. Labium (Figs. 4g, q, 5c, d) with submentum trapezoid, fused with gula, deflected and forming angle with the basis of mentum. Mentum slightly convex, deeply depressed anteriorly, dorsolateral fold with large fovea, disc with about four lateral long setae and with one transverse strong keel. Prementum membranous; palpiger prominent and with ventroproximal asperites; glossa (gls) club-like and with five distal setae; paraglossa (pgl) blade-like with outer setae-like cuticular projections; palp with three palpomeres. Hypopharynx (Fig. 5c) covered with dense setae-like cuticular projections, anterior area with a transverse row of about 15 large sensilla. Antenna with ten antennomeres; scape as wide as pedicel, and slightly wider than funicle antennomeres; antennal club with proximal flagellomere longer than medial one, and medial flagellomere longer than distal one. *Prothorax* (Figs. 7a–d): Pronotum wider than long, entirely beaded, marginal bead with long yellow or testaceous setae, surface slightly convex, sparsely punctate and setose, interpuncture smooth and shiny; pronotal scar as a shallow concavity; anterior angles acute, anteriorly prominent; posterior angles rounded. Hypomeron with posterior arm with two carinate articular process (Fig. 7d). Prosternum with basisternum anteriorly prominent and medially with longitudinal carina; posterior sternal process slightly acuminate. Cervix (Fig. 7a) with three sclerites, two ventrolateral and one minor dorsolateral. *Pterothrorax* (Figs. 8, 9a–d): Mesothoracic spiracle about 2.5 times longer than wide (Fig. 7b), metathoracic spiracle about 3.3 times longer than wide (Fig. 9d). Mesoscutellum (Figs. 9a, b) triangular, laterals slightly arcuate, surface punctate and setose as pronotum, separate from scutum by a rounded deflection. Mesepisternum (Figs. 9c, d) with anterior ventral angle prominent, metepisternum (Fig. 8e) separate from mesocoxal cavity by the mesepimeron-metaventrite contact. Mesoventrite with posterior area separate mesocoxae cavity, deflected and forming an angle of 120º with the anterior half in lateral view (Fig. 8e). Metaventrite longer at the middle, punctate and setose, posterior margin sinuous, medial posterior area pointed backward. Metendosternite (Fig. 8b, c, f) with large basis and anterior area prominent. *Elytra*: Outer, distal and inner margins beaded; interstriae granulated and setose; nine punctate stria present, I–V at disc, inner to humerus and separate from each other by about 3 punctures diameter, VI–IX at lateral and outer to humerus and separate from each other by less than one puncture diameter; inner posterolateral surface with longitudinal carina; posterior angle slightly prominent. Posterior wings (Fig. 9e–l): Anterior margin fused with radial cel forming club-like area formed by costa (C), subcosta (Sc) and anterior sector of radius (RA); basis of fourth anterior radial vein (RA_4_) and basis of first posterior radial vein (RP_1_) indistinct; third and fourth posterior medial veins (MP_3_ and MP_4_) distinct; distal part of anterior cubital vein (CuA) distally bent; radial fulcalare distinct as slightly sclerotized band basally in contact with second axillary sclerite (Fig. 9f, j); head of first axillary sclerite narrow (Fig. 9g–i); dorsal distal ridge of second axillary sclerite short (Fig. 9j, k); caudal area of third axillary sclerite slightly shorter than cranial anterior area (Fig. 9l). *Legs*: Profemur with inner carina between trochanter articulation and posterior femur-tibia articulation. Protibiae (Fig. 2g, 7e–g) with three outer teeth; inner distal angle not prominent, rounded; males with posterior distal area with two tubercles each with one thin seta (Fig. 7e, g), females with tubercles inconspicuous and bearing one thick seta (Fig. 7f), posterior proximal and medial area with a row of four to eight acute teeth. Mesocoxae oblique, posteriorly convergent; widely separate from each other (Fig. 2e). Anterior side of meso- and metafemora with four longitudinal rows of long setae. Metafemora inner margin crenulate or with one small tooth. Mesotibiae shorter than mesotarsomeres I–V combined; surface covered by spines and setae. Metatibiae with three longitudinal rows of spine-like setae. Meso- and metatarsomere I slightly dilated; longer than tarsomeres II–IV combined. Longest mesotibial spur spiny serrate. *Abdomen* (Figs. 10, 11): Sternites III–VIII ventrally exposed; II thin, with medial lamellate process (ps2), dorsal fold small and trapezoid. Surface of sternites III–VIII smooth, covered by yellow setae; dorsal fold of sternite III (cs3) with anterior cavity as long as the length of fold (Fig. 10c); dorsal fold of sternite VII with small club-like stridulatory peg (sp; Fig. 10a). Propygidium with spiracles at anterior angles (as7); elytral interlocking apparatus formed by two posterior teeth, medial area without process or carina (Figs. 2f, 10a). Pygidium (pyg) densely punctate and setose; broad triangular, anterior angles with spiracles; anterior margin with transversal shallow sulcus (Fig. 10c). Male terminalia (Figs. 10e–g) flat and tubular; tergite IX (t9) medially interrupted, posterolateral lobes setose, and with each anterolateral area ventrally bent, fold as a thin bar anterior to sternite IX; sternite IX long, with posterior area setose. Aedeagus (Fig. 10h–p) with short and almost symmetrical parameres. Female terminalia (Fig. 11a–c) with paraprocts (t9; Fig. 11f) long, with posterior inner membranous punctate area; each side of external genitalia with shield-like punctate proximal gonocoxite (ppg; Fig. 11h); small medial gonocoxite (mpg) bearing two setae (long and minute; Fig. 11i, j); distal club-like setose gonocoxite (dpg; Figs. 11k–n); and small distal gonostyle (stl) bearing three distal setae. Internal genitalia (Fig. 11d–e) with long bursa copulatrix (bcx) connected to left side of duct, with some long thick straight setae at basis; spermatheca somewhat oblong (spt; Fig. 11o–r); gland of spermatheca (gst) small, connected to basis of spermatheca; accessory glands indistinct.

#### **Sexual dimorphism**

Males and females are easily separated from each other specially by the ornaments of protibia. Males of *Atlantochodaeus* present protibial spur strongly bent (Figs. 2g, 7e), while it is slightly curved in females (Fig. 7f). Males with posterior side of protibia with two distal tubercle-like process, each bearing one thin seta; otherwise, females have inconspicuous processes, and the setae associated with each process are thick. Also, mentum presents the truncated transverse keel (Fig. 4g, q) that is relatively larger in males than in females. Metafemora ornaments (Fig. 13) smaller in females than in males, sometimes indistinct.

#### **Remarks**

Female terminalia of *Atlantochodaeus* is similar to that found in other Ochodaeinae, but the medial gonocoxite is smaller (Fig. 11i, j) than in *Ochodaeus* species (Dupuis 2005).

#### **Type-species**

*Atlantochodeus everardoi* **n. sp.**, here designated.

#### **Geographic distribution**

**Fig. 14:**
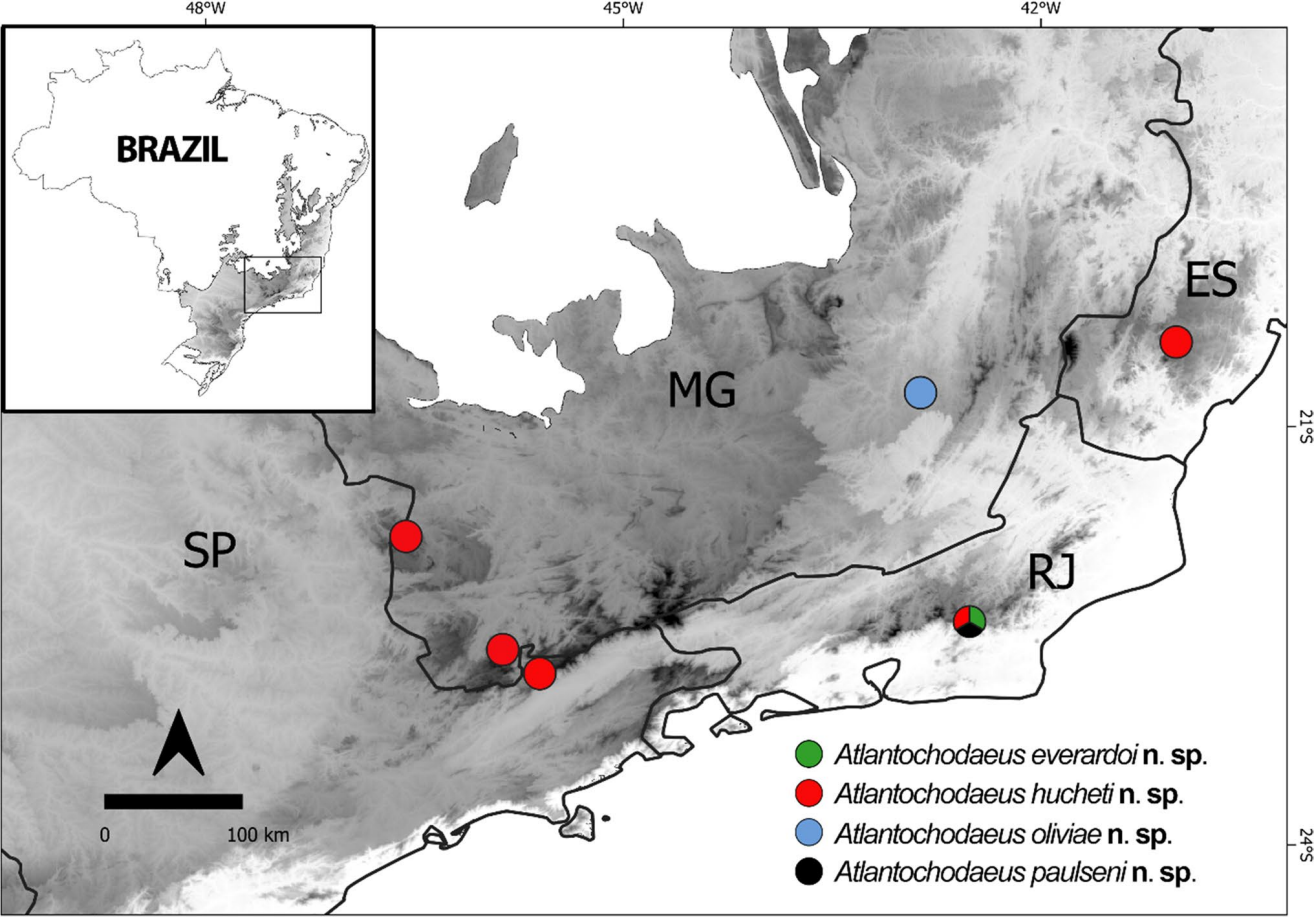
Occurrence of *Atlantochodaeus* species from the Brazilian Atlantic Forest: *A*. *everardoi ***n. sp.** (grey green circle), *A*. *hucheti* **n. sp.** (black red circle), *A*. *oliviae* **n. sp.** (black rhombusblue circle), and *A*. *paulseni* **n. sp.** (white black circle). Legend of Brazilian States: ES = Espírito Santo; MG = Minas Gerais; and RJ = Rio de Janeiro; and SP = São Paulo

Atlantic Forest of Southeast Brazil (Fig. 14).

#### **Etymology**

The name is derived from “*Atlant*-” (referring to the Atlantic Forest), the geographical origin of the species comprising the new genus, combined with the generic name “*Ochodaeus*”; gender masculine.

*Atlantochodaeus everardoi* **n. sp.**

(Figs. 1a–d, 2a–g, 3e–h, 4a–j, 5, 6, 7a–f, 7h, 8, 9, 10a–c, 10e–j, 11a–i, k, o, 12, 13a, 14).

(urn:lsid:zoobank.org:act:32D27 C46-A50D-413B-87 A8-A7 C929DB623 F).

#### Type material

**Holotype, male** (Fig. 1a–c). Labels (Fig. 1d), first label [white, printed]: “BRASIL: Rio de Janeiro,/Nova Friburgo, Macaé de/Cima, ii.2006, E.J. Grossi.”. Second label [red with black frame, printed]: “♂/*Atlantochodaeus*/*everardoi*/des. Costa-Silva et al./HOLOTYPE/2024” **(CERPE)**. **Type locality**. Macaé de Cima, Nova Friburgo municipality, Rio de Janeiro State, Brazil.

#### **Paratypes (40 specimens)**

First label [white, printed]: “BRASIL: Rio de Janeiro,/Nova Friburgo, Macaé de/Cima, ii.2006, E.J. Grossi.”. Second label [yellow with black frame]: “*Atlantochodaeus*/*everardoi*/des. Costa-Silva et al./PARATYPE/2024” **(1 female [MZSP 22217], MZSP)**. First label [white, printed]: “BRASIL: RJ/Nova Friburgo/III-1998 FIT/P. and E. Grossi”. Second label [yellow with black frame]: “*Atlantochodaeus*/*everardoi*/des. Costa-Silva et al./PARATYPE/2024” **(1 male [MZSP 22216], MZSP)**. First label [white, printed]: "BRASIL, RJ, Nova Friburgo/Macaé de Cima, RPPN/Bacchus, iii.2007, 1500 m/E. and P. Grossi leg". Second label [yellow with black border, printed]: “*Atlantochodaeus*/*everardoi*/des. Costa-Silva et al./PARATYPE/2024” **(1 female, CERPE).** First label [white, printed]: "Coleção E./and P. Grossi". Second label [white, printed]: “BRASIL, RJ, Nova/Friburgo, Macaé de/Cima, 1300 m, I.2010 E.J. Grossi.”. Third label [yellow with black frame]: “*Atlantochodaeus*/*everardoi*/des. Costa-Silva et al./PARATYPE/2024” (**1 male, CEMT**; **1 male and 2 females, CERPE**). First label [white, printed]: “BRASIL, Rio de Janeiro, Nova/Friburgo, Macaé de cima,/1500 m, xii.2009, FIT,/P.J. Grossi leg”. Second label [white, unknown’s handwriting] “M.C. XII.09/FIT”. Third label [yellow with black frame]: “*Atlantochodaeus*/*everardoi*/des. Costa-Silva et al./PARATYPE/2024” (**1 male, CERPE**). First label [white, printed]: “BRASIL: Rio de Janeiro, Nova Friburgo, Macaé de/Cima, ii.2006, E.J. Grossi.” Second label [yellow with black frame]: “*Atlantochodaeus*/*everardoi*/des. Costa-Silva et al./PARATYPE/2024” (**2 males and 2 females, CEMT; 2 males and 2 females, CERPE**). First label [white, printed]: “BRASIL: Rio de/Janeiro, Nova Fribur/go, Macaé de Cima,/x.2000, P. Grossi”. Second label [yellow with black frame]: “*Atlantochodaeus*/*everardoi*/des. Costa-Silva et al./PARATYPE/2024” (**1 female, CEMT**). First label [white, printed]: “BRASIL: Rio de Janeiro/Nova Friburgo, Macaé/de Cima I-1999 PGrossi.”. Second label [yellow with black frame]: “*Atlantochodaeus*/*everardoi*/des. Costa-Silva et al./PARATYPE/2024” (**2 males and 2 females, CEMT, 2 females, CERPE**). First label [white, unknown’s handwriting]: “BRASIL: RJ/Nova Friburgo/Macaé de Cima/IV-2000/P. and E. Grossi”. Second label [yellow with black frame]: “*Atlantochodaeus*/*everardoi*/des. Costa-Silva et al./PARATYPE/2024” (**2 males, CEMT; 2 males, CERPE**). First label [white, printed]: “BRASIL: RJ, Nova Friburgo/Macaé de Cima XII-2000/P and E Grossi legs”. Second label [yellow with black frame]: “*Atlantochodaeus*/*everardoi*/des. Costa-Silva et al./PARATYPE/2024” (**1 male, CEMT; 1 male CERPE**). First label [white, printed]: “BRASIL, RJ, Nova Friburgo/Macaé de Cima, III-2000/1390 m, I.V. [Interception Trap], I. Miller col./22º22′31”S/42º29′45”W”. Second label [white, printed]: “COLEÇÃO/E., and P. Grossi”. Third label [yellow with black frame]: “*Atlantochodaeus*/*everardoi*/des. Costa-Silva et al./PARATYPE/2024” (**1 male and 1 female, CEMT; 2 males and 1 female, CERPE**). First label [white, printed]: “BRASIL: RJ Nova Friburgo/Macaé de Cima 1500 m/III-2000 Lopes-Andrade, Gumier and Vaz-de-Mello”. Second label [yellow with black frame]: “*Atlantochodaeus*/*everardoi*/des. Costa-Silva et al./PARATYPE/2024” (**1 male, CEMT**). First label [white, printed]: “Macaé de Cima/Nova Friburgo/RJ—BRASIL/I-2006/Leg: B.Miller”. Second label [yellow with black frame]: “*Atlantochodaeus*/*everardoi*/des. Costa-Silva et al./PARATYPE/2024″ (**1 male, CEMT**). First label [white]: “Macaé de Cima/Nova Friburgo/RJ—Brasil/I—2006/Leg: B. Miller”. Second label [yellow with black frame]: “*Atlantochodaeus*/*everardoi*/des. Costa-Silva et al./PARATYPE/2024″ (**3 males and 1 female, AMBC**).

#### **Differential diagnosis**

*Atlantochodaeus everardoi* is similar to *A*. *hucheti* and both share the body mainly black, metafemur with inner area crenulate (Fig. 13a–b), and male protibial spur abruptly bent (as in Figs. 2g and 7e). *Atlantochodaeus everardoi* can be separated by following characteristics (*A*. *hucheti* morphology between square brackets): pronotum reddish or yellowish brown (Figs. 1a, 12) [pronotum dark reddish brown, almost black, Fig. 1e]; metafemur inner area weakly crenulate (Fig. 13a) [strongly, Fig.  13b]; female spermatheca somewhat ovoid (Fig. 11o) [oblong, Fig. 11Q].

#### **Holotype**

(Figs. 1a–c) length: 6.8 mm, width: 3.9 mm, body dark reddish brown, almost black; pronotum reddish brown; setae yellow or black. *Head*. Eyes very small in ventral view (head 1.4 wider than minor ventral interocular distance; Fig. 3g). Fronto-clypeal suture complete. Mentum with curved keel, U-shaped in ventral view (similar to Fig. 4g). Prementum with anterior area lobed (Fig. 4h). *Thorax*. Pronotum length: 2.1 mm, width: 3.4 mm. *Elytra*: Length: 3.7 mm, width: 3.9 mm. *Legs*: Protibiae with posterior proximal and medial areas with row of six acute teeth (Fig. 7e). Metafemur narrow with inner area slightly crenulate (Fig. 13a). Metatibia inner side arched, without emargination (Fig. 7h). Aedeagus with anterior half of parameres abruptly bent in lateral view (Figs. 10h–j).

#### **Variations**

This species presents a great variability in length and colour (Fig. 12), being the body black or reddish brown, pronotum yellowish and reddish brown, elytra with or without a small or large light macula. Body length: 5.3–7.0 mm, width: 2.9–4.0 mm. Pronotum can be reddish brown or yellowish brown; and elytra with or without light macula, small in humerus (Fig. 12c) or large in basal area (Fig. 12d). Protibiae with posterior proximal and medial areas with a row of four to six acute teeth. Females with spermatheca somewhat ovoid (Fig. 11o).

#### **Geographic distribution**

Known from Serra de Macaé de Cima, Nova Friburgo municipality, Rio de Janeiro (Fig. 14).

#### **Etymology**

It is a noun in a genitive case (masculine), named after the great clinical pathologist and enthusiast entomologist, Dr. Everardo J. Grossi, for all his wonderful contribution and incentive for the study of beetles’ fauna, and for collecting most of the specimens here studied.

*Atlantochodaeus hucheti* **n. sp.**

(Figs. 1e‒h, 10 k– l, 11 l, p, 13b).

(urn:lsid:zoobank.org:act:FF97612 A-69D9-42B9-B9DD-9 F5 F0833462 C).

#### Type material

**Holotype, male** (Fig. 1e–g). Labels (Fig. 1h), first label [white, printed]: “BRASIL: ES 1500 m [meters above sea level]/P.E. [State Park] Pedra Azul/I – 2000 Lopes-Andrade/and Vaz-de-Mello legs.”. Second label [red with black frame, printed]: “♂/*Atlantochodaeus*/*hucheti*/des. Costa-Silva et al./HOLOTYPE/2024” **(CEMT)**. **Type locality**. Parque Estadual da Pedra Azul, Domingos Martins municipality, Espírito Santo State, Brazil.

#### **Paratypes (24 specimens)**

First label [white, printed]: “BRASIL: ES 1500 m [meters above sea level]/P.E. [State Park] Pedra Azul/I – 2000 Lopes-Andrade/and Vaz-de-Mello legs.”. Second label [yellow with black frame, printed]: “*Atlantochodaeus*/*hucheti*/des. Costa-Silva et al./PARATYPE/2024” **(4 males and 3 females, CEMT; 5 males and 2 females, CERPE; 1 male [MZSP 22215] and 1 female [MZSP 22214], MZSP)**. First label [white, printed]: “BRASIL: RJ/Nova Friburgo/XI 1998/P. and E. Grossi”. Second label [yellow with black frame, printed]: “*Atlantochodaeus*/*hucheti*/des. Costa-Silva et al./PARATYPE/2024” **(1 male, CEMT)**. First label [white]: “Pedra Azul—1500 m./Domingos Martins/ES—Brasil/I—2000/Col: A. Bello”. Second label [yellow with black frame, printed]: “*Atlantochodaeus*/*hucheti*/des. Costa-Silva et al./PARATYPE/2024” (**1 male and 1 female, CERPE**). First label [white, printed]: “BRASIL, São Paulo, Campos do/Jordão (hotel Toriba),/22°46′15’’S 45°35′52’’,/17–20.ii.2025, L. J. Migliore, G. Biffi,/J. Furhmann, T. Silva, G, Fiuza leg.”. Second label [yellow with black frame, printed]: “*Atlantochodaeus*/*hucheti*/des. Costa-Silva et al./PARATYPE/2024” (**1 female, CEMT; 1 male [MZSP 22211]and 1 female [MZSP 22212], MZSP**). First label [white, printed]: “BRASIL – Minas Gerais/Poços de Caldas/Morro S. Domingos/15–19.5.1968/ J. Becker/O. Roppa e O. Leoncini cols.”. Second label [white, printed]: “Coleção/Vulcano”. Third label [yellow with black frame, printed]: “*Atlantochodaeus*/*hucheti*/des. Costa-Silva et al./PARATYPE/2024” **(1 male [MZSP 22213], MZSP)**. First label [white aged with black frame]: “J. Zikán [vertical]/Passa Cuatro/915 m/S. Minas Ger./17.ii.1923/Serra/dos/côchos/1400 m”. Second label [white aged]: “Coleção/J. F. Zikan”. Third label [yellow with black frame, printed]: “*Atlantochodaeus*/*hucheti*/des. Costa-Silva et al./PARATYPE/2024” (**1 male, CEIOC**).

#### **Differential diagnosis**

See *A*. *everardoi* above.

#### **Holotype**

(Fig. 1e–g) length: 6.6 mm, width: 3.7 mm, body reddish brown, almost black, elytra bearing a basal yellow macula (1/4 of entire length). *Head*. Eyes very small in ventral view (head 1.4 wider than minor ventral interocular distance). Fronto-clypeal suture complete. Mentum with curved keel, U-shaped in ventral view. Prementum with anterior area lobed (similar to Fig. 4h). *Thorax*. Pronotum length: 1.8 mm, width: 3.4 mm. *Elytra*: Length: 3.2 mm, width: 3.6 mm. *Legs*: Protibiae with posterior proximal and medial areas with row of six acute teeth, (similar to Fig. 7e). Metafemur narrow with inner area strongly crenulate (Fig. 13b). Metatibia inner side arched, without emargination (similar to Fig. 7h). Aedeagus with parameres slightly curved in lateral view (Figs. 10k–l).

#### **Variations**

Body length: 6–7.5 mm, width: 3.0–4.1 mm. This species presents variation in body length and colour, with specimens being either black or reddish brown. The basal yellow macula of the elytra also varies in shape and size, ranging from a small spot in the humeral area to a large macula that may cover more than half of the elytra. Protibiae with posterior proximal and medial areas with a row of five to six acute teeth. Females with spermatheca oblong (Fig. 11p).

#### **Geographic distribution**

Known from Serra de Macaé de Cima, Nova Friburgo municipality (RJ), Serra do Castelo, Domingos Martins municipality (ES), Campos do Jordão municipality (SP), and Morro S. Domingos, Poços de Caldas municipality (MG) (Fig. 14).

#### **Etymology**

It is a noun in a genitive case (masculine), named after the colleague and dear friend, Dr. Jean-Bernard Huchet (Muséum national d’Histoire naturelle, France), for is contribution to the taxonomy of the Ochodaeidae fauna from Old World and other families of Scarabaeoidea.

*Atlantochodaeus oliviae* **n. sp.**

(Figs. 1i–l, 4k–t, 7g, i, 10m–n, 11j, m, q, 13c)

(urn:lsid:zoobank.org:act:21375704-5 C9E-42 AE-9664-36D0BE0708 A1)

#### Type material

**Holotype, male** (Fig. 1i–k). Labels (Fig. 1l), first label [white, typeset]: “BRASIL: MG/Viçosa FIT/Vaz-de-Mello/XII-98 [vertical]”. Second label [red with black frame, printed]: “♂/*Atlantochodaeus*/*oliviae*/des. Costa-Silva et al./HOLOTYPE/2024” **(CEMT)**. **Type locality**. Viçosa municipality, Minas Gerais State, Brazil.

#### **Paratypes (15 specimens)**

First label [white, typeset]:** “**BRASIL: MG/Viçosa FIT/Vaz-de-Mello/XII-98 [vertical]”. Second label [yellow with black border, printed]: “*Atlantochodaeus*/*oliviae*/des. Costa-Silva et al./PARATYPE/2024” **(1 male and 1 female, CEMT; 2 males and 2 females, CERPE; 1 male [MZSP 22219] and 1 female [MZSP 22218], MZSP)**. First label [white, typeset]:** “**BRASIL: MG/Viçosa FIT/Vaz-de-Mello/II-1999 [vertical]”. Second label [yellow with black border, printed]: “*Atlantochodaeus*/*oliviae*/des. Costa-Silva et al./PARATYPE/2024” **(1 male and 1 female, CEMT; 2 males CERPE)**. First label [white, typeset]:** “**BRASIL: Minas/Gerais; Viçosa/XII-2000 FIT [flight interception trap]/Vaz-de-Mello”. Second label [yellow with black border, printed]: “*Atlantochodaeus*/*oliviae*/des. Costa-Silva et al./PARATYPE/2024” **(2 females** [one specimen without abdomen] **CEMT)**. First label [white, typeset]:** “**BRASIL: MG, Viçosa/Mata do Paraíso/19.ii.2015; FIT [flight interception trap]/leg. S. Aloquio, A. Orsetti and M. Bento”. Second label [yellow with black border, printed]: “*Atlantochodaeus*/*oliviae*/des. Costa-Silva et al./PARATYPE/2024” **(1 male, CEMT)**.

#### **Differential diagnosis**

*Atlantochodaeus oliviae* is similar to *A*. *paulseni* and both share the body yellowish color, metafemur with an inner tooth or lobe, and male protibial spur gradually bent. *Atlantochodaeus oliviae* can be separate by following characteristics (*A*. *paulseni* morphology between square brackets): mentum with curved keel (Fig. 4q), inverted U-shaped in ventral view [U-shaped]; metafemora wide and with inner tooth near the base (Fig. 13c) [narrow and with small lobe, Fig. 13d]; metatibiae without inner emargination (Fig. 7h) [with weak emargination, Fig. 7i]; female spermatheca somewhat sinuous (Fig. 11q) [oblong, Fig. 11r].

#### **Holotype**

(Figs. 1i–k) length: 9.2 mm, width: 4.8 mm, body yellowish brown. *Head*: Eyes small in ventral view (head 1.6 wider than minor ventral interocular distance). Fronto-clypeal suture medially indistinct. Mentum with curved keel, inverted U-shaped in ventral view (Fig. 4q). Prementum with anterior area emarginate (Fig. 4r). *Thorax*: Pronotum length: 2.8 mm, width: 4.4 mm. *Elytra*: Length: 5.4 mm, width: 4.8 mm. *Legs*: Protibiae with posterior proximal and medial areas with row of seven acute teeth (Fig. 7g). Metafemora wide with inner area slightly strait and bearing small lobe (Fig. 13c). Metatibia inner side arched and with small emargination (Fig. 7i). Aedeagus with parameres slightly curved in lateral view (Fig. 10m–n).

#### **Variation**

Body light or dark yellowish brown, length: 6.3–9.4, width: 3.5–5.4. Protibiae with posterior proximal and medial areas with row of six to eight acute teeth. Females with spermatheca sinuous (Fig. 11q).

#### **Sexual dimorphism**

The females of *Atlantochodaeus oliviae* **n. sp.** present the sternites of the abdomen completely rounded at the centre, while is flat in males.

#### **Geographic distribution**

Known from Viçosa municipality, MG (Fig. 14).

#### **Etymology**

It is a name in a genitive case (feminine), named in honour of Olívia da Costa Leipnitz, the young niece of the first author, who has always motivated him with all her brilliance and love.

*Atlantochodaeus paulseni* **n. sp.**

(Fig. 1m–o, 10o–p, 11n, r, 13d)

(urn:lsid:zoobank.org:act:D048D95B-840D-475E-BABB-8153B3BA7D4 C)

#### **Type material**

**Holotype, male** (Fig. 1m–o). Labels (Fig. 1p), first label [white, printed]: “BRASIL, RJ, Nova Friburgo/Macaé de Cima, V-2007/1390 m [meters above sea level], I.V. [Interception Trap], I. Miller col./22º22′31”S/42º29′45″W”. Second label [red with black frame, printed]: “♂/*Atlantochodaeus*/*paulseni*/des. Costa-Silva et al./HOLOTYPE/2024″ (**CERPE**). **Type locality**. Macaé de Cima, Nova Friburgo municipality, Rio de Janeiro State, Brazil.

#### **Paratypes (5 specimens)**

First label [white, printed]: “BRASIL, RJ, Nova Friburgo/Macaé de Cima, V-2007/1390 m, I.V. [Interception Trap], I. Miller col./22º22′31”S/42º29′45”W”. Second label [yellow with black border, printed]: “*Atlantochodaeus*/*paulseni*/des. Costa-Silva et al./PARATYPE/2024” (**1 male, CEMT; 1 female [MZSP 22220], MZSP**). First label [white, printed]: “BRASIL, RJ, Nova/Friburgo, Macaé de/Cima, 1300 m, I.2010/E.J. Grossi Leg”. Second label [yellow with black border, printed]: “*Atlantochodaeus*/*paulseni*/des. Costa-Silva et al./PARATYPE/2024″ (**1 female, CEMT**). First label [white, printed]: "BRASIL, RJ, Nova Friburgo/Macaé de Cima, RPPN/Bacchus, iii.2007, 1500 m/E. and P. Grossi leg". Second label [yellow with black border, printed]: “*Atlantochodaeus*/*paulseni*/des. Costa-Silva et al./PARATYPE/2024″ (**1 male and 1 female, CERPE**).

#### **Differential diagnosis**

See *A*. *olivae* above.

#### **Holotype**

(Fig. 1m–o) length: 7.5 mm, width: 3.9 mm, body yellowish brown. *Head*: Eyes small in ventral view (head 1.6 wider than minor ventral interocular distance). Fronto-clypeal suture medially indistinct. Mentum with sightly curved keel, U-shaped in ventral view. Prementum with anterior area lobed (similar to Fig. 4h). *Thorax*: Pronotum length: 1.7 mm, width: 3.3 mm. *Elytra*: Length: 4.5 mm, width: 3.9 mm. *Legs*: Protibiae with posterior proximal and medial areas with a row of six acute teeth, (similar to Fig. 7g). Metafemora wide with inner area slightly straight and bearing one small tooth (Fig. 13d). Metatibia inner side arched, without emargination. Aedeagus with parameres slightly curved in lateral view (Fig. 10o–p).

#### **Variation**

Body light or dark yellowish brown, length: 7.8–8.6 mm, width: 4.1–4.5 mm. Protibiae with posterior proximal and medial areas with row of six to seven acute teeth. Females with spermatheca oblong (Fig. 11r).

#### **Geographic distribution**

This species is known from Serra de Macaé de Cima, Nova Friburgo municipality, RJ (Fig. 14).

#### **Etymology**

It is a name in a genitive case (masculine), named after the great entomologist Dr. Matthew J. Paulsen (University of Nebraska, USA), for all his contribution to the study of Ochodaeidae and other Scarabaeoidea fauna around the World.

## Key to South American Ochodaeidae genera and species of *Atlantochodaeus*

**1** – Meso- and metatibial spurs pectinate, metatibia somewhat flat, and abdomen without stridulatory apparatus….*Gauchodaeus* Paulsen 2012 (Chaetocanthinae).

**1’** – One mesotibial spur pectinate, metatibia not flat, and abdomen usually with stridulatory apparatus…2 (Ochodaeinae).

**2** – Eyes prominent (Fig. 3b) and wide in ventral view (head 2.5 wider than minor ventral interocular distance; Fig. 3c), with posterior margin emarginate (Fig. 2k); mesocoxae subcontiguous (Fig. 2l); apex of elytra strongly prominent (Fig. 2m)….***Parochodaeus*** Nikolajev 1995

**2’** – Eyes not prominent (Fig. 3f, j) and very small in ventral view (head 1.4–1.6 wider than minor ventral interocular distance; Fig. 3g, k), with posterior margin rounded (Fig. 2d); mesocoxae widely separate (Fig. 2e, white arrow); apex of elytra slightly prominent (Fig. 2m) … ***Atlantochodaeus* n. gen**…3

**3** – Body yellowish brown, eye small in ventral view (Fig. 3k); frontoclypeal suture medially indistinct, laterals of suture distinct and dark, male protibial spur gradually bent, inner area of metatibia crenulate (Fig. 13a, b)…4

**3’** – Body dark reddish brown, almost black, with pronotum and elytral humerus light or not, eye very small in ventral view (similar to 3 g), frontoclypeal suture entire, male protibial spur abruptly bent (Fig. 2g), inner area of metatibia lobed or toothed (Fig. 13c, d)…5

**4** – Mentum with an inverted U-shaped keel (Fig. 4q); metafemur wide and with an inner tooth (Fig. 13c); metatibiae with weak inner emargination (Fig. 7i)….*Atlantochodaeus oliviae* **n. sp.**

**4’** – Mentum with a U-shaped keel (similar to Fig. 4g); metafemur narrow and with a small inner lobe (Fig. 13d); metatibiae without emargination (similar to Fig. 7h)….*Atlantochodaeus paulseni* **n. sp.**

**5** – Pronotum reddish or yellowish brown (Figs. 1a, 12); inner area of metatibia weakly crenulate (Fig. 13a)….*Atlantochodaeus everardoi* **n. sp.**

**5’** – Pronotum black or dark reddish brown, almost black (Fig. 1e); inner area of metatibia strongly crenulate (Fig. 13b)….*Atlantochodaeus hucheti* **n. sp.**

## Discussion

### Tribal Placement

*Atlantochodaeus* is placed into tribe Ochodaeini based on follows characteristics: mandibles and pedicel small (large in Endognathini), scutellum short with posterior angle slightly rounded (long and acuminate in Endognathini); female with gonostyle (absent in Notochodaeini); and propygidial stridulatory apparatus bearing two small posterior teeth (similar to *Parochodaeus*, Ochodaeini). Regarding other genera of the tribe (see introduction), *Parochodaeus* and *Atlantochodaeus* are similar to each other, and both have a toothed propygidium stridulatory apparatus (see apparatus discussion above).

*Atlantochodaeus* shares with Nothochodaeini genera a few morphological characteristics, such as the medium to large size (5–12 mm in Nothochodaeini and 6–8 mm in *Atlantochodaeus*) and mesocoxae widely separated (Fig. 2e, white arrow). Bi-colored species (as *Atlantochodaeus everardoi* and *A*. *hucheti*) is also frequent in Nothochodaeini species (see Huchet 2022); the coloration is uniform reddish-brown or testaceous, from light to dark in other genera of Ochodaeidae (Paulsen 2012).

### Stridulatory Apparatus

The stridulatory mechanism of Ochodaeidae is formed mainly by a dorsal peg of sternite VI (*pars stridens*; Fig. 10a–d) and a longitudinal stria on the inner side of the elytra (*plectrum*). Possibly the interlock apparatus of propygidium help to hold elytra in correct position to the sound production, and the lateral concavity of sternite III (cs3; Fig. 10c, d; hidden by closed elytra) is probably a kind of resonance chamber (Arrow 1904; Paulsen 2019; Huchet 2020). Interestingly, the interlocking apparatus of propygidium and the stridulatory peg are both absent in the Ochodaeini genus *Cucochodaeus*, showing evidence to the relation of interlocking apparatus and the stridulatory system. Also, is important to note that the club-like anterior margin of posterior wings, is placed between elytral stridulatory stria and the sternite stridulatory peg; and the ventral surface of radial cel has some relatively long asperites, but not forming an evident delimited area; maybe the posterior wing also play a role in stridulation on Ochodaeidae. The structure of the interlocking apparatus and stridulatory system of *Atlantochodaeus* (Figs. 2f, 10c) is quite similar to those of *Parochodaeus* (Figs. 2m, 10c), but all pieces are relatively smaller in the former, including the size of the sternite concavity. This variation certainly shows that the sound produced is different to each taxon. Further studies are needed to clarify the mechanism of sound production and bioacoustics of these scarab beetles.

### Glands and Mycangium

Two conspicuous characteristics of *Atlantochodaeus* and *Parochodaeus* possibly related to glands are present: mandibular glands and terminalia glands.

The dorsal side of mandibles of the genera have small punctures that are connected to ducts with a basal large ovoid structure that resembles gland (detail of Fig. 6a). Mandibles’glands are rarely reported to scarab beetles; some Allidiostomatinae (Scarabaeidae) probably have glands that are opened in the ventral area of mandibles (Frolov 2012).

Female terminalia of scarab beetles present usually two or four accessory glands and a spermatheca glad (Dupuis 2005). The accessory glands of *Atlantochodaeus*, *Parochodaeus* and *Ochodaeus* (see Dupuis 2005) are indistinct, but a membranous area of paraprocts and proximal gonocoxites have some puncture connected to ducts with a basal branched structure that resembles glands (present in *Atlantochodaeus* species and *P*. *pectoralis*; Fig. 11g). Accessory glands are often absent in some Geotrupidae (Dupuis 2005).

Mandibles of the genera have some tiny and shallow concave structures (detailed in Fig. 6b) on the dorsal surface. These structures could be mycangia, with the punctuations possibly containing fungal cells. Symbiotic relationships between Scarabaeoidea and fungi have been reported in several families, including Lucanidae, Geotrupidae, Passalidae, Ochodaeidae, and Scarabaeidae (Carlson and Ritcher 1974; Rahola-Fabra 2004; Tanahashi et al. 2010; Miquel and Vasko 2014; Rosa-Gruszecka et al. 2017).

Information regarding the biology and feeding habits of ochodaeids is scarce. Carlson and Ritcher (1974) were one of the first to suggest mycetophagous feeding habits in Ochodaeidae after discovering small basidiomycete spores in the mid- and hindgut contents of some species. Furthermore, the association of Ochodaeidae with hypogeous fungi reported by Huchet et al. (2022) supports the theory of mycetophagous feeding habits, shedding light on the hypothesis of the potential mycangium presence.

The true nature of structures such as glands and mycangium in Ochodaeidae remains poorly understood. Further studies are needed to a better clarification and comprehension of these structures.

## Data Availability

Data generated or analysed during this study are provided in full within the published article.
